# Proportion of children with cancer that have an indication for genetic counseling and testing based on the cancer type irrespective of other features

**DOI:** 10.1007/s10689-021-00234-4

**Published:** 2021-02-26

**Authors:** Thi Minh Kha Nguyen, Astrid Behnert, Torsten Pietsch, Christian Vokuhl, Christian Peter Kratz

**Affiliations:** 1 Pediatric Hematology and Oncology, Hannover Medical School, Carl-Neuberg-Str. 1, 30625 Hannover, Germany; 2 Department of Neuropathology, Institute of Neuropathology, Brain Tumor Reference Center, University of Bonn, Bonn, Germany; 3 Section of Pediatric Pathology, Institute of Pathology, University Hospital Bonn, Bonn, Germany

**Keywords:** Cancer predisposition syndromes, Pathology, Childhood cancer

## Abstract

In children with cancer, specific clinical features such as physical anomalies, occurrence of cancer in young relatives, specific cancer histologies, and unique mutation/methylation signatures may indicate the presence of an underlying cancer predisposition syndrome (CPS). The proportion of children with a cancer type suggesting a CPS among all children with cancer is unknown. To determine the proportion of children with cancer types suggesting an underlying CPS among children with cancer. We evaluated the number of children with cancer types strongly associated with CPS diagnosed in Germany between 2007 and 2016. Data were obtained from various sources including two national pediatric pathology reference laboratories for brain and solid tumors, respectively, various childhood cancer trial offices as well as the German Childhood Cancer Registry. Among 21,127 children diagnosed with cancer between 2007 and 2016, 2554 (12.1%) had a cancer type strongly associated with a CPS. The most common diagnoses were myelodysplastic syndrome and juvenile myelomonocytic leukemia, retinoblastoma, malignant peripheral nerve sheath tumor, infantile myofibromatosis, medulloblastoma^SHH^, rhabdoid tumor as well as atypical teratoid/rhabdoid tumor. Based on cancer type only, 12.1% of all children with cancer have an indication for a genetic evaluation. Pediatric oncology patients require access to genetic counselling and testing.

## Introduction

Cancer predisposition syndromes (CPS) are a well-established cause of childhood cancer [1–4]. Several clinical features may prompt a physician to consider an underlying CPS in a child with cancer. These include physical anomalies, positive cancer (family) history, and cancer type as well as somatic molecular features [4–7]. While there are cancer types rarely associated with a CPS, such as acute lymphoblastic leukemia (ALL), germ cell tumors, and neuroblastoma, there are other cancer types that, by itself, always suggest the presence of an underlying CPS. Examples include pleuropulmonary blastoma strongly associated with *DICER1* germline variants or retinoblastoma strongly associated with pathogenic variants in *RB1*. Here, we evaluated the proportion of children with cancer types strongly associated with CPS diagnosed within a ten-year, 2007–2016, period in Germany. Addressing this question is important in order to better estimate genetic counseling needs in the field of pediatric oncology.

## Methods

Cancer types with an indication for genetic counseling and testing were recently reviewed and incorporated in a CPS-questionnaire currently used in Germany to identify patients with an indication for a genetic evaluation (see supplement to Ref. [6]). The list of cancer types falling into this category was created by the cancer predisposition working group of the German Society for Pediatric Oncology and Hematology and was based on recommendations from various cancer trial groups. The list shares large overlaps with a similar list generated independently by Postema et al. [4] who created a list of cancer types that warrant a genetic evaluation because at least 5% of cases are associated with a CPS. The number of patients with the following cancer types diagnosed in Germany between January 1st 2007 and December 31st 2016 were retrieved from the pediatric cancer pathology reference laboratories of the German Society of Oncology and Hematology (GPOH) in Bonn where generally all cases of childhood tumors are reviewed centrally: adrenocortical adenoma, adrenocortical carcinoma, atypical teratoid/rhabdoid tumor, botryoid rhabdomyosarcoma of the genitourinary tract, choroid plexus carcinoma, colorectal carcinoma, cystic nephroma, fetal rhabdomyoma, gastrointestinal stromal tumor, giant cell glioblastoma, gonadoblastoma, hemangioblastoma, hepatocellular carcinoma, infantile myofibromatosis, large cell calcifying Sertoli-cell-tumor, malignant peripheral nerve sheath tumor, medullary renal cell carcinoma, medullary thyroid carcinoma, medulloepithelioma, melanoma, meningioma, myxoma, neuroendocrine tumor (excluding carcinoid of the appendix), optic pathway glioma, parathyroid adenoma, pheochromocytoma, pilocytic astrocytoma with signs of neurofibromatosis type 1, pineoblastoma, pituitary adenoma/tumor, pleuropulmonary blastoma, renal cell carcinoma, rhabdoid-tumor, anaplastic rhabdomyosarcoma, schwannoma, Sertoli-Leydig-cell-tumor, small cell carcinoma of the ovary hypercalcemic type, squamous cell carcinoma, subependymal giant cell astrocytoma, thyroid carcinoma (follicular), thyroid carcinoma (papillary), thyroid carcinoma (unclassified). In addition, the number of patients with the following cancer types diagnosed within the same 10-year period were retrieved from the annual report of the German Childhood Cancer Registry (GCCR) [8]: myelodysplastic syndrome/juvenile myelomonocytic leukemia, and retinoblastoma.

The number of patients diagnosed in Germany within the same 10-year period with the following entities were calculated based on numbers provided in the annual report of the GCCR [8] and the published percentage of cases with the defining features [9–12]: medulloblastoma^SHH^, medulloblastoma^WNT^ *CTNNB1* wildtype, hepatoblastoma *CTNNB1* wildtype and acute myeloid leukemia (AML) with monosomy 7/aberration 7q. Finally, the number of patients diagnosed with hypodiploid ALL was provided by the ALL BFM and COALL trial groups (C.P.K. personal communication).

Beginning in 2009, the GCCR extended the upper age cut-off from 15 years to 18 years. According to the GCCR’s archived annual reports, 16,902 patients under the age of 18 years were diagnosed between years 2009 and 2016 (on average, 2112 patients per year). Based on this number, we estimate that 21,127 patients under the age of 18 years were diagnosed with cancer between January 1st 2007 and December 31st 2016. The study was approved by the ethical review board at Hannover Medical School.

## Results

**Fig. 1:**
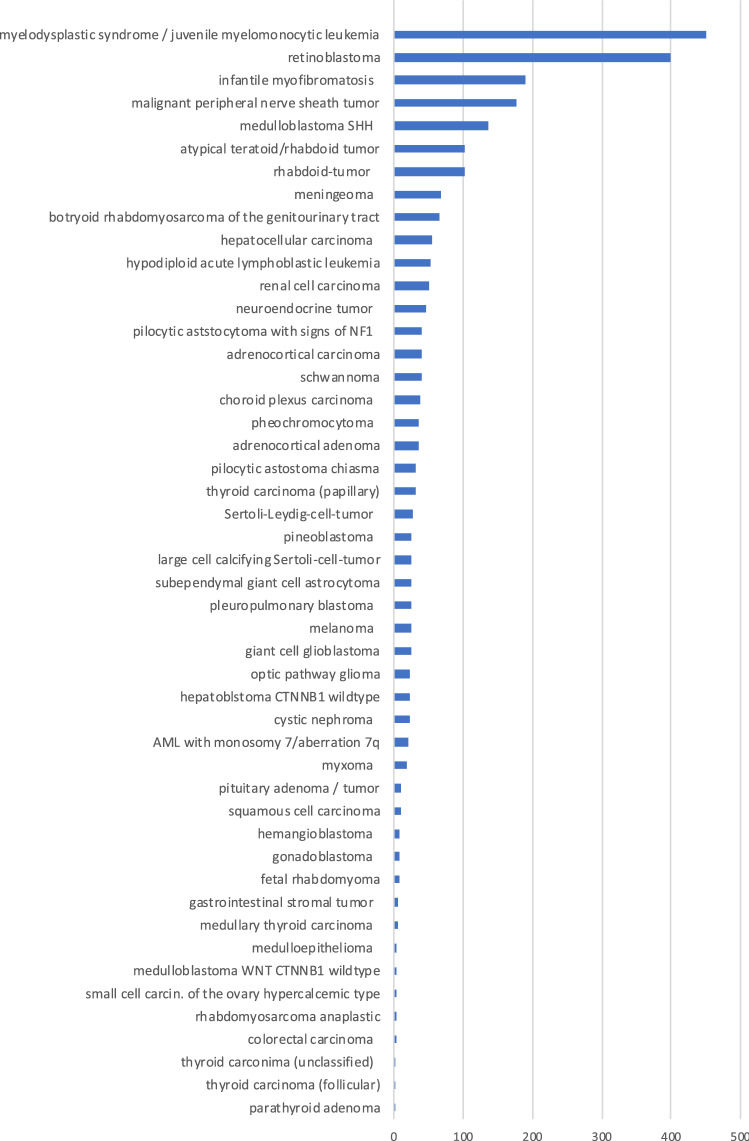
Number of children with various cancer types highly associated with cancer predisposition syndromes diagnosed in Germany within a 10-year period, 2007–2016

Among the 21,127 patients diagnosed with cancer prior to age 18 years in Germany between January 1st 2007 and December 31st 2016, 2554 children (12.1%) had a cancer type highly suggesting the presence of an underlying CPS. The most common diagnoses were myelodysplastic syndrome, juvenile myelomonocytic leukemia, retinoblastoma, malignant peripheral nerve sheath tumor, infantile myofibromatosis, medulloblastoma^SHH^, rhabdoid tumor and atypical teratoid/rhabdoid tumor. For all the remaining entities there were less than 10 patients per year diagnosed in Germany (Fig. 1).

## Discussion

Approximately 10% of children with cancer have a CPS [1–4]. Therefore, a CPS should be, at least, considered, in every child with cancer. The optimal approach to diagnose children with a CPS among children with cancer is currently being investigated by several groups. One strategy is to clinically identify individual patients who have a particular high probability of an underlying CPS and to offer genetic evaluation to these individuals. We and others have established simple tools in order to help physicians to identify patients with an indication for genetic counseling and testing [4–7]. In addition to family history and physical anomalies, the cancer type (including somatic signatures) may help to identify patients with a high probability of an underlying CPS. The cancer types falling into this category are constantly growing and evolving [13]. Here, we determined the proportion of children with cancer types highly suggestive of an underlying CPS and found that 14% of children with cancer fall into this category.

The study has several limitations: (1) The study design did not allow us to determine the percentage of patients who had an underlying CPS; It is likely that many patients were never genetically evaluated because in the past patients were not studied systematically; (2) We cannot rule out that we missed patients because our main identifying sources were two reference laboratories. It is possible that not all patients with relevant cancer types were reviewed by these reference laboratories. Nevertheless, the use of pathology reference laboratories is a common practice in Germany and generally, most pediatric cases are reviewed. We cannot rule out, however, that all pathologies from children with “adult” tumor types (e.g. colorectal carcinoma) were reviewed by one of the pediatric reference laboratories that served as our main source; (3) not all tumors are biopsied (e.g., optic pathway glioma in patients with neufibromatosis type 1 are not always biopsied) potentially leading to an underestimation in this study. (4) The cancer types associated with CPS are constantly evolving. For example, after our data collection was completed, *TRIM28* pathogenic variants were identified in epithelial nephroblastoma [14–17]. Thus, our list of cancer types is incomplete, leading to an underestimation of the determined proportion; (5) Several cancer types, especially brain tumors, are now subdivided into molecular subgroups. Only recently it has been shown that some of these subgroups are often associated with a CPS and, therefore, these data were not available for the entire 10-year period. We were able to compensate this problem by providing estimates in this situation (see "Methods" section).

In conclusion, 12.1% of children with cancer have a cancer type strongly associated with a CPS. In order to increase the chance that affected families are being offered counseling and testing, reference pathology reports on these entities should state that a genetic evaluation is recommended. We also recommend that pediatric oncologists use one of the available screening tools [4–7]-some of which use decisional algorithms [7]-to identify patients with a high likelihood of an underlying CPS.

## Data Availability

Raw data and material and processed data are held within the Department of Pediatric Hematology and Oncology at Hannover Medical School.
